# Beyond Isotretinoin: A Narrative Review of Emerging Systemic Therapies for Moderate‐to‐Severe Acne

**DOI:** 10.1111/jocd.70812

**Published:** 2026-03-31

**Authors:** Nello Tommasino, Maria Carmela Annunziata, Luca Potestio, Maddalena Napolitano

**Affiliations:** ^1^ Section of Dermatology, Department of Clinical Medicine and Surgery University of Naples Federico II Naples Italy

**Keywords:** acne, management, systemic therapies

## Abstract

**Background:**

Acne vulgaris is a common inflammatory skin disorder with significant psychosocial impact. While isotretinoin remains the gold standard for moderate‐to‐severe cases, its adverse effects and teratogenicity underscore the need for alternative systemic therapies.

**Objective:**

To review emerging systemic treatments for acne beyond isotretinoin, evaluating their efficacy, safety, and potential clinical applications.

**Methods:**

A narrative review was conducted through PubMed, Scopus, and Web of Science, including studies from January 2015 to June 2025. Eligible publications focused on systemic treatments for moderate‐to‐severe acne, excluding topical‐only and non‐peer‐reviewed studies.

**Results:**

Promising alternatives include the narrow‐spectrum antibiotic sarecycline, hormonal agents such as spironolactone and combined oral contraceptives, and metformin in patients with polycystic ovary syndrome. Biologic therapies, phosphodiesterase‐4 inhibitors, oral probiotics, and novel agents like montelukast and CAMP factor vaccines are also under investigation. These treatments show varying degrees of efficacy and improved safety profiles, but most lack head‐to‐head comparisons with isotretinoin.

**Conclusion:**

Several systemic options beyond isotretinoin have emerged, showing potential in specific subgroups or as adjunctive therapies. However, further large‐scale comparative studies are needed to define their roles within evidence‐based acne management algorithms.

## 1 Introduction

Acne vulgaris is a skin condition characterized by comedones, papules, pustules, and nodules and is one of the most common inflammatory diseases [1]. Specifically, it is estimated that 85% of people between the ages of 12 and 29 have suffered from it [2]. In relation to the age of onset, it is classified into young acne (10–24 years old) and specifically into adolescent acne if the age is between 10 and 19 years old and into adult acne from the age of 25 years old [1]. Although the estimate is controversial, generally a slightly higher incidence of young acne in males is reported, while adult acne is significantly more common in females [1]. Several factors contribute to the pathogenesis of acne, such as bacterial proliferation of Cutibacterium (C.) acnes, increased sebum secretion, altered follicular keratinization, and hyperandrogenism [3]. It affects the face in most cases and can cause permanent scarring. For these reasons, it can easily have a negative emotional impact on affected patients. In fact, acne is associated with low self‐esteem, embarrassment, social anxiety, and isolation [4]. The goals of acne treatment are to control lesions, prevent scarring, and limit the duration and impact of the disease on the patient [4]. Treatment options include topical and systemic drugs, chosen according to the severity and appearance of the pathology. The former includes retinoids (e.g., adapalene, tazarotene), antibiotics (clindamycin, erythromycin), benzoylperoxide (BPO), and others [4]. Traditional systemic drugs, on the other hand, include antibiotics such as azithromycin, tetracyclines, clindamycin, and isotretinoin as a retinoid. The latter is used for severe nodular acne or in cases resistant to other topical and oral therapies [4]. However, both oral antibiotics and isotretinoin present important limitations. Long‐term antibiotic use contributes to bacterial resistance and may alter the microbiome, while isotretinoin, though highly effective, requires close monitoring due to teratogenicity, mucocutaneous side effects, and potential other adverse events. For this reason, the development of new systemic treatments that are safer, better tolerated, and more targeted is a growing need in dermatological practice. In addition to efforts aimed at optimizing existing antibiotic classes, recent research has focused on identifying novel antimicrobial targets against Cutibacterium acnes. These approaches move beyond conventional inhibition of protein synthesis and include strategies targeting bacterial membrane integrity, ion channel regulation, and selective antimicrobial peptides, with the aim of reducing resistance development and microbiome disruption. There are also several complementary physical therapies that are also useful for reducing acne scars, such as CO2 lasers and chemical peeling [4]. Due to the frequency of the condition, the high negative impact on patients, and the limitations of the currently available systemic therapies, it is necessary to implement the therapeutic armamentarium against acne. The objective of this manuscript is to review both emerging systemic therapies and established systemic alternatives that may represent valuable options in selected patient profiles beyond isotretinoin.

## 2 Materials and Methods

This narrative literature review was conducted to identify and analyze recent evidence on systemic treatments for acne vulgaris beyond isotretinoin. A comprehensive search was performed using databases including PubMed, Scopus, and Web of Science, covering articles published from January 2015 to June 2025. The following keywords were used: “acne vulgaris”, “systemic treatment”, “oral treatment”, “beyond isotretinoin”, “non‐isotretinoin therapies”, “new treatments”. Inclusion criteria were: original research articles, clinical trials, systematic reviews, or meta‐analyses; studies focused on systemic treatments for moderate to severe acne; and publications in English. Exclusion criteria included case reports, non‐peer‐reviewed articles, and studies focused exclusively on topical therapies or cosmetic approaches. Reference lists of relevant articles were also screened to identify additional studies. The collected data were analyzed to summarize current knowledge on emerging systemic therapies, such as hormonal agents, antibiotics with novel mechanisms, selective androgen receptor inhibitors, and biologics. Results are briefly summarized in Table 1.

**TABLE 1:** Summary of emerging systemic treatments for moderate‐to‐severe acne beyond isotretinoin. The table includes the main therapeutic classes, their mechanisms of action, supporting clinical evidence, and relevant clinical considerations.

Therapy/Class	Target/Mechanism	Evidence summary	Notes
**Sarecycline**	Narrow‐spectrum tetracycline, inhibits protein synthesis	Phase III trials: effective in facial/truncal acne; QoL improvement	Low phototoxicity; approved ≥ 9 years
**Oral zinc**	Anti‐inflammatory, reduces C. acnes lipase, immunomodulatory	Open‐label study non‐inferior to lymecycline	Low cost, stewardship‐oriented alternative
**Pentobra**	Peptide‐aminoglycoside, disrupts membrane & ribosomes	Preclinical; superior to tobramycin in vitro	Not yet approved
**p‐Carboethoxy derivatives**	Targets mechanosensitive channels in C. acnes	In vitro/in vivo inhibition of C. acnes growth	Experimental stage only
**Adalimumab**	Anti‐TNF‐α monoclonal antibody	Case reports in acne conglobata and SAPHO	Off‐label; small series only
**Apremilast**	PDE‐4 inhibitor, reduces inflammation	Case reports in acne fulminans and SAPHO	Off‐label use in refractory cases
**Metformin**	Reduces IGF‐1, improves insulin resistance	Meta‐analysis in PCOS; RCTs show lesion reduction	Particularly effective in women with PCOS
**Spironolactone**	Anti‐androgen, blocks androgen receptors	RCTs and meta‐analysis confirm efficacy in women	Effective, but off‐label and limited male data
**Combined oral contraceptives**	Suppresses androgenic hormonal effects	Comparable to antibiotics by 6 months	Risk of thromboembolism; avoid in high‐risk patients
**Oral probiotics**	Modulates immune response, increases IL‐10	Several RCTs show reduced lesions, especially adjunctive	Promising as adjunct, not stand‐alone
**Montelukast**	CysLT1 receptor antagonist, anti‐inflammatory	RCTs show efficacy alone or with doxycycline	Needs more data; well tolerated

Abbreviations: IGF‐1, Insulin‐like Growth Factor 1; PCOS, Polycystic Ovary Syndrome; PDE‐4, Phosphodiesterase type 4; QoL, Quality of Life; RCT, Randomized Controlled Trial; TNF‐α, Tumor Necrosis Factor alpha.

## 3 Results

### 3.1 Antibiotic Drugs

The increasing awareness of antibiotic resistance in C. acnes has stimulated the exploration of innovative antimicrobial strategies targeting previously unexploited bacterial structures. Among these, mechanosensitive ion channels of large conductance (MscL) have emerged as promising targets. The p‐carboethoxy‐tristyrylbenzene derivative class of antibiotics has demonstrated, in both in vitro studies and murine models, significant inhibition of C. acnes growth [5, 6]. Pentobra is a peptide‐aminoglycoside molecule that has shown in vitro selectivity in killing C. acnes without acting against human cells. It acts by combining the mechanism of antimicrobial peptides and aminoglycosides. In fact, it increases bacterial membrane permeability and blocks the activity of their ribosomes, respectively. Pentobra also demonstrated greater bactericidal performance than tobramycin in a direct comparison [7]. Sarecycline is a narrow‐spectrum tetracycline specifically developed to minimize activity against enteric Gram‐negative flora while maintaining efficacy against C. acnes. In two identically designed phase III trials (SC1401 and SC1402), sarecycline demonstrated significant superiority over placebo in reducing inflammatory lesions as early as week 3, with sustained improvement in both facial and truncal acne up to week 12 [8, 9]. Investigator Global Assessment (IGA) success rates approached 60% at week 12, accompanied by measurable improvements in patient‐reported outcomes, including Skindex‐16 and ASIS scores [8, 10]. Safety findings were particularly notable. Adverse events were mostly mild (headache, nasopharyngitis, nausea), treatment discontinuation occurred in only 2.5% of patients, and phototoxic reactions, commonly associated with other tetracyclines, were reported in ≤ 1% of cases [9, 11]. This favorable tolerability profile, together with its selective antimicrobial spectrum, suggests a reduced impact on the gut microbiome compared with conventional tetracyclines, positioning sarecycline as a more stewardship‐oriented systemic antibiotic option for acne management.

Baldwin et al. evaluated the change from baseline in ASIS responses after 12 weeks. Among 253 patients, the mean ASIS score decreased significantly for signs, social impact, and emotional impact [10]. The IGA was also evaluated, with a success rate of 58.9% [10]. Moore et al. also used ASIS to evaluate the performance of sarecycline on truncal acne of 10 patients, adding 10 questions focused on their concerns (ASIS‐C). The mean ASIS‐C score decreased by 4% for signs, 15% for impactions, and 16% for concerns, with greater increases in those patients who had achieved complete or nearly complete clearance of truncal acne [12]. Regarding trunk acne, Del Rosso et al. performed a pooled analysis of the SC1401 and SC1402 trials, showing significantly higher rates of IGA success than the placebo group for chest and back. In the former case, the IGA success rates after 3 weeks were 11.84% and 7.71% for sarecycline and placebo groups respectively and then increased to 18.81% vs. 14.03% after 6 weeks respectively and 33.42% vs. 20.77% after 12 weeks respectively [13]. For back acne, the rates were 12.13% vs. 7.04%, 18.42% vs. 14.34%, and 33.07% vs. 21.91% at 3, 6, and 12 weeks respectively [13]. An additional systemic option aligned with principles of antibiotic stewardship is oral zinc therapy. Zinc salts have long been recognized for their anti‐inflammatory, sebostatic, and antimicrobial properties, and are endorsed in several international acne treatment guidelines as an alternative to oral antibiotics in selected patients. Zinc modulates neutrophil chemotaxis, inhibits C. acnes lipase activity, and reduces pro‐inflammatory cytokine release, contributing to lesion improvement without contributing to antibiotic resistance [14]. Clinical evidence supports its efficacy. In an open‐label comparative study, Tolino et al. demonstrated that oral zinc was non‐inferior to lymecycline in reducing inflammatory lesion counts over 12 weeks of treatment, with a more favorable safety profile and absence of microbiome disruption [14]. These findings position oral zinc as a low‐cost, well‐tolerated systemic alternative, particularly suitable for patients in whom long‐term antibiotic use is undesirable.

### 3.2 Biologic Drugs

Beyond its classical classification as a disorder of the pilosebaceous unit, acne is increasingly recognized as an immune‐driven inflammatory disease. C. acnes activates innate immunity through Toll‐like receptors, leading to the production of pro‐inflammatory cytokines such as IL‐1β, TNF‐α, and IL‐17. The Th17 axis, inflammasome activation, and sustained neutrophilic infiltration play a central role in the development of severe inflammatory lesions, particularly in nodulocystic and conglobate forms. Within this framework, anti‐TNF‐α and anti‐IL biologics have been explored in particularly severe or syndromic acne phenotypes. In acne conglobata, the use of the anti‐Tumor Necrosis Factor‐α (TNF‐α) adalimumab has been reported [15, 16]. A new approach described by Ping et al. combines adalimumab with photodynamic therapy (PDT) with 5‐Aminolaevulinic acid, used on an 18‐year‐old patient with severe acne treated without benefit with isotretinoin and systemic antibiotics. The authors administered adalimumab every 2 weeks for 3 times and performed PDT once a week for 3 weeks. The lesions improved already after the first week of combination therapy until resolution in the following weeks [17]. Yang et al. also reported the success of adalimumab on a case of SAPHO (synovitis, acne, pustulosis, hyperostosis, and osteitis) syndrome concomitant with palmoplantar pustulosis [18]. Several case reports describe SAPHO treated with the anti‐interleukin (IL)‐23 risankizumab [19] and the anti‐Il‐17 secukinumab [20, 21]. Thiboutot et al. however, demonstrated in a trial of anti‐IL‐17A CJM112 no statistically significant difference between this drug, similar to secukinumab in mechanism of action, and placebo on the reduction of moderate‐to‐severe acne inflammatory lesions [22]. Although evidence is currently limited to case reports and small series, these experiences support the concept that selected acne phenotypes may represent inflammatory endotypes potentially amenable to targeted immunomodulation.

### 3.3 Phosphodiesterase‐4 Inhibitors

Apremilast is a phosphodiesterase (PDE)‐4 inhibitor approved for psoriasis, whose anti‐inflammatory activity has been considered for use in severe forms of acne. Sánchez‐Velázquez et al. used it in a 15‐year‐old patient who developed acne fulminans of the trunk and back during oral isotretinoin therapy. After scaling down isotretinoin and introducing systemic corticosteroids, the patient improved but as the dose of corticosteroid was reduced, he developed flare‐ups of the pathology. The authors started apremilast at the recommended dose for psoriasis, with marked improvement after 6 weeks and achievement of skin clearance after 10 months [23]. Adamo et al. also reported the case of a SAPHO syndrome treated with apremilast in a 24‐year‐old patient refractory to previous biological therapies of different classes [24].

### 3.4 Metformin

Although not a novel drug, metformin has gained increasing attention as a valuable systemic alternative for acne in selected metabolic and hormonal contexts, particularly in women with polycystic ovary syndrome (PCOS) and insulin resistance. Hyperandrogenism is among the predisposing factors for the occurrence of acne. It causes abnormal stimulation of hyperkeratinization and increased secretion of sebaceous glands [25]. Women with polycystic ovary disease (PCOS) have elevated levels of insulin‐like growth factor‐1 (IGF‐1), which stimulates the ovary's production of androgen hormones [25]. Consequently, women with PCOS have forms of acne that are resistant to conventional therapies. These patients may benefit from metformin therapy, which reduces IGF‐1 levels [25]. A meta‐analysis by Yen et al. of 51 studies and 2405 PCOS patients proved that metformin leads to improvements in acne both as monotherapy and as adjuvant therapy [26]. In this regard, Robinson et al. on a group of 84 acne patients treated with tetracycline 250 mg bd and topical BPO 2.5% compared those who also received metformin 850 mg daily and those who did not. After 12 weeks there was an average percentage improvement in lesions of 71.4% in the metformin group and 65.3% in the other [27]. The metformin group also showed a treatment success rate of 66.7%, compared with 43.2% for the other [27]. Sharma et al. showed significant improvement in 40 women with PCOS and acne treated with metformin 500 mg 3 times daily as early as 3 weeks of therapy, with results maintained until week 8 [28]. Similarly, another study of 39 patients with PCOS treated in the same way observed a statistically significant improvement in acne score of 14% [29]. In contrast, Fabbrocini et al. investigated the action of metformin on 20 male patients with acne and altered metabolic profile. Among them, 10 patients received metformin 500 mg bd associated with a low‐calorie diet and the remaining did not. After 6 months, a statistically significant improvement in acne was observed in the former group [30].

### 3.5 Oral Probiotics

Oral probiotics are considered in the treatment of acne because of their immune‐modulating properties and ability to raise levels of the anti‐inflammatory cytokine IL‐10 [31] and decrease the pro‐inflammatory cytokine IL‐8 [32]. A double‐blind placebo‐controlled study of 20 patients showed significant improvement in acne among 10 patients who had received *Lactobacillus rhamnosus* GG for 12 weeks, compared with the placebo group [33]. Skin biopsies of participants taken before and after treatment were also compared, which demonstrated reduction in IGF‐1 levels [33]. Rinaldi et al. evaluated the effects of a dietary supplement of different probiotics on 114 acne‐moderate patients demonstrating a significant reduction in inflammatory lesions, sebum secretion rate, and desquamation score after 8 weeks, associated with high tolerability [34]. Marasca et al. confirmed the promising therapeutic role of oral probiotics in a study of 30 patients over 60 days of therapy in which they combined these with topical therapy with azelaic acid, hydroxypinacolone retinoate, and α‐hydroxy acids. At the follow‐up visit, in fact, improvement was observed in all scores considered with no adverse effects reported [35]. Jung et al. instead compared 45 patients divided into groups that received probiotic supplementation, oral minocycline alone, or were treated with both. After 12 weeks of therapy, the group receiving minocycline and probiotics had significantly greater outcomes than the other two groups. The authors suggest the potential role of probiotics in combination with other systemic therapies for more severe forms of acne [36].

### 3.6 Hormonal Therapies

Hormonal therapies are not emerging treatments per se but represent well‐established systemic alternatives for acne in female patients with androgen‐driven disease. Their relevance in the context of this review lies in their role as effective non‐isotretinoin systemic options tailored to specific patient profiles. A growing body of evidence supports the efficacy of spironolactone, an oral anti‐androgen, in managing moderate to severe acne in females. A recent meta‐analysis involving 1086 patients showed that oral spironolactone at 100 mg/day significantly reduced total lesion count by a mean difference (MD) of −6.85 and improved Acne Severity Index (ASI) by MD −6.33 compared to placebo, with favorable safety outcomes [37]. Another meta‐analysis confirmed its effectiveness with a pooled odds ratio (OR) of 2.51 for treatment success versus placebo or doxycycline [38]. Data from randomized controlled trials demonstrate that spironolactone outperforms doxycycline in adult women—after 6 months, treatment success rates were 2.87 times higher (*p* = 0.007), alongside quality‐of‐life improvements [39]. Combined oral contraceptives (COCs) also play a substantial role. Trials show they are as effective as oral antibiotics by 6 months, reducing both inflammatory and non‐inflammatory lesions. Common side effects include breast tenderness, breakthrough bleeding, and increased risk of thromboembolism in high‐risk populations [40].

### 3.7 Experimental and Future Directions

Among experimental approaches currently under investigation, vaccine‐based strategies targeting C. acnes represent a promising but still preclinical field of research. Liu et al. developed an antibody neutralizing the CAMP factor of C. acnes and demonstrated, in murine models, a reduction in bacterial colonization without affecting commensal flora [41]. At present, these findings remain limited to animal studies and no clinical data in humans are available. Although no acne‐specific data exist, vaccine‐induced exacerbation of inflammatory dermatoses has been reported in other contexts [42]. This observation represents a theoretical concern that should be considered in the future development of acne‐targeted immunotherapies.

Rokni et al. showed the efficacy of oral montelukast, a CysLT1 receptor antagonist, in treating acne, with the severity score decreasing from 33.93 at baseline to 20.6 among 30 patients after 12 weeks of therapy [43]. Behrangi et al. compared montelukast with oral doxycycline in the treatment of moderate acne in 52 patients. A significant reduction in acne lesions was observed in both groups, with no statistically significant differences [44]. Fazelzadeh‐Haghighi et al. demonstrated with a study of 108 patients with moderate acne the superiority of montelukast 10 mg/day combination with doxycycline 100 mg/day over doxycycline alone, with improvement in all scores and acne lesion counts [45]. These approaches should be regarded as long‐term research directions rather than clinically applicable options at present.

## 4 Discussion

Emerging systemic therapies for acne reflect a movement toward mechanism‐driven treatments offering safety and antimicrobial stewardship advantages over isotretinoin. Isotretinoin remains the most effective systemic therapy: network meta‐analyses consistently identify isotretinoin as the top‐ranking systemic agent for moderate‐to‐severe acne, achieving around a 58% mean reduction in lesions at high cumulative dosing (≥ 120 mg/kg) and strong odds of treatment success versus placebo and other therapies [46]. Its impact is broad, targeting sebum production, follicular hyperkeratinization, inflammation, and *C. acnes* colonization. This potency comes at the cost of frequent mucocutaneous side effects, laboratory abnormalities, strict teratogenic control programs, but also severe AEs such as acne fulminans [47]. By contrast, sarecycline demonstrates moderate efficacy with significant reductions in inflammatory lesions as early as 3 weeks and sustained improvement through 12 weeks, plus quality‐of‐life benefits and low rates of phototoxicity and microbiome disruption. However, without direct trials comparing it to isotretinoin or conventional tetracyclines, definitive conclusions about its relative efficacy or cost‐effectiveness are limited [48]. According to a network meta‐analysis of 221 randomized clinical trials, oral isotretinoin is confirmed as the most effective treatment for acne on all counts. Oral Sarecycline was shown to be significantly superior to placebo in treating inflammatory acne lesions, but not significantly superior on non‐inflammatory lesions [49]. In this context, oral zinc therapy represents a particularly valuable option, combining anti‐inflammatory efficacy with absence of antibiotic resistance pressure and excellent tolerability. This further reinforces the concept of antibiotic stewardship discussed in this review. Biologic and immunomodulatory agents and novel anti‐inflammatory approaches (e.g., apremilast, case‐based use of adalimumab) reflect an inclination toward targeting acne's immune dysregulation. While anecdotal successes in acne conglobata and SAPHO syndrome offer proof‐of‐concept, the failure of IL‐17A inhibition (CJM112) [22] in controlled trials underscores heterogeneity in inflammatory endotypes and cautions against generalizing efficacy without biomarkers guiding patient selection. Hormonal therapies, specifically spironolactone, are supported by robust evidence in adult women. A recent meta‐analysis of RCTs (seven studies, 643 participants) showed spironolactone significantly reduced the ASI versus placebo, with a mean difference of −6.53, indicating a clinically meaningful effect on overall lesion count and severity [50]. In trials, most AEs were mild: headaches, dizziness, and menstrual irregularities. No serious AEs or discontinuations due to hyperkalemia, a described AE with spironolactone, were reported [50]. This, the lack for teratogenic risk and not intensive laboratory surveillance are significant advantages over isotretinoin. Despite these strengths, its off‐label status and lack of male‐focused trials or long‐term safety data represent notable limitations. The lack of comparative studies between isotretinoin and spironolactone is another important limitation to quantifying the effectiveness of hormone therapies. Oral probiotics leverage microbiome‐based immunomodulation to reduce inflammatory lesions and sebum production, showing promise as adjunctive therapies especially in antibiotic‐intolerant patients. Still, limited standardization, strain variability, and small sample sizes undermine reproducibility and broader applicability. Importantly, not all therapies discussed in this review are “emerging” in a chronological sense. Agents such as metformin and spironolactone represent established systemic treatments whose relevance lies in their ability to serve as effective, targeted alternatives to isotretinoin in specific hormonal and metabolic patient subsets. Metformin is effective in reducing acne severity, particularly in PCOS patients, through mechanisms like reduced IGF‐1, improved insulin resistance, and mTORC1 inhibition [51]. Tolerability and metabolic benefits of metformin contrast with isotretinoin related AEs and teratogenic concerns. Isotretinoin remains superior, especially at low doses, but metformin offers a viable alternative in women, particularly those with PCOS, by simultaneously targeting metabolic factors and acne [52]. Beyond efficacy and safety, practical considerations significantly influence the real‐world applicability of several therapies discussed in this review. Biologic agents and immunomodulators, while mechanistically appealing, are associated with substantial costs, limited reimbursement, and are currently supported only by off‐label evidence in acne. Similarly, drugs such as apremilast may present regulatory and access barriers that restrict their use to highly selected, refractory cases. Even for agents with stronger evidence, such as spironolactone and metformin, off‐label prescribing and the absence of formal acne indications in many countries may limit their routine adoption. These economic and regulatory factors must be considered when translating emerging or alternative systemic therapies into clinical practice, as they represent significant barriers alongside scientific evidence.

This review has several strengths. It provides an updated and wide‐ranging synthesis of systemic acne therapies from multiple pharmacological domains, including newer agents under investigation. By covering both clinical trial data and real‐world experiences, it offers a comprehensive landscape of treatment alternatives that could be tailored to individual patient profiles. Moreover, it emphasizes the importance of moving toward a personalized and mechanism‐based approach in acne management. However, important limitations must be acknowledged. As a narrative review, it does not follow systematic review methodology and lacks quantitative synthesis, which may introduce selection bias. Many treatments discussed, especially biologics, apremilast, and montelukast, are supported only by small studies or case reports, limiting generalizability. In addition, the absence of head‐to‐head trials against isotretinoin hinders accurate comparative positioning of these therapies, and heterogeneity across studies in patient populations and outcome measures complicates the interpretation of efficacy and safety. Future research should focus on well‐designed randomized controlled trials, comparative effectiveness studies, and biomarker‐guided approaches to better define the role of these emerging therapies within modern acne treatment algorithms.

## 5 Conclusions

Isotretinoin remains the most effective systemic therapy for moderate‐to‐severe acne, but its side effect profile and teratogenic risk necessitate safer, targeted alternatives. Emerging options, including sarecycline, spironolactone, metformin in PCOS, biologics, and oral probiotics, demonstrate promising efficacy and improved tolerability in selected populations. However, the lack of large‐scale, head‐to‐head trials with isotretinoin limits their integration into treatment algorithms. Future studies should prioritize comparative research and biomarker‐driven approaches to support a more personalized and mechanism‐based management of acne.

## Funding

The authors have nothing to report.

## Ethics Statement

The authors have nothing to report.

## Consent

The authors have nothing to report.

All authors read and approved the final version of the manuscript.

## Conflicts of Interest

The authors declare no conflicts of interest.

## Data Availability

Data that support the findings of this study are available from the corresponding author, upon reasonable request.
